# Enhanced efficacy of combined temozolomide and bromodomain inhibitor therapy for gliomas using targeted nanoparticles

**DOI:** 10.1038/s41467-018-04315-4

**Published:** 2018-05-18

**Authors:** Fred C. Lam, Stephen W. Morton, Jeffrey Wyckoff, Tu-Lan Vu Han, Mun Kyung Hwang, Amanda Maffa, Elena Balkanska-Sinclair, Michael B Yaffe, Scott R Floyd, Paula T Hammond

**Affiliations:** 1 Koch Institute for Integrative Cancer Research, Massachusetts Institute of Technology, Cambridge, MA 02139 USA; 2 Center for Precision Cancer Medicine, Massachusetts Institute of Technology, Cambridge, MA 02139 USA; 3 Department of Chemical Engineering, Massachusetts Institute of Technology, Cambridge, MA 02139 USA; 4 Marble Center for Cancer Nanomedicine, Massachusetts Institute of Technology, Cambrige, MA 02139 USA; 5 Department of Radiation Oncology, Duke University School of Medicine, Durham, NC 27710 USA; 6 Department of Pharmacology and Cancer Biology, Duke University School of Medicine, Durham, NC 27710 USA; 7 Departments of Biology and Bioengineering, Massachusetts Institute of Technology, Cambridge, MA 02139 USA; 8 Department of Surgery, Beth Israel Deaconess Medical Center, Harvard Medical School, Boston, MA 02215 USA

## Abstract

Effective treatment for glioblastoma (GBM) is limited by the presence of the blood–brain barrier (BBB) and rapid resistance to single agent therapies. To address these issues, we developed a transferrin-functionalized nanoparticle (Tf-NP) that can deliver dual combination therapies. Using intravital imaging, we show the ability of Tf-NPs to traverse intact BBB in mice as well as achieve direct tumor binding in two intracranial orthotopic models of GBM. Treatment of tumor-bearing mice with Tf-NPs loaded with temozolomide and the bromodomain inhibitor JQ1 leads to increased DNA damage and apoptosis that correlates with a 1.5- to 2-fold decrease in tumor burden and corresponding increase in survival compared to equivalent free-drug dosing. Immunocompetent mice treated with Tf-NP-loaded drugs also show protection from the effects of systemic drug toxicity, demonstrating the preclinical potential of this nanoscale platform to deliver novel combination therapies to gliomas and other central nervous system tumors.

## Abstract (web summary)

The blood-brain barrier often limits effective delivery  of treatments for glioblastoma . In this study, the authors develop transferrin-functionalized nanoparticles able to traverse the intact blood-brain barrier and deliver combination temozolomide and bromodomain inhibitor therapy to  glioma-bearing mice.

## Introduction

**Fig. 1:**
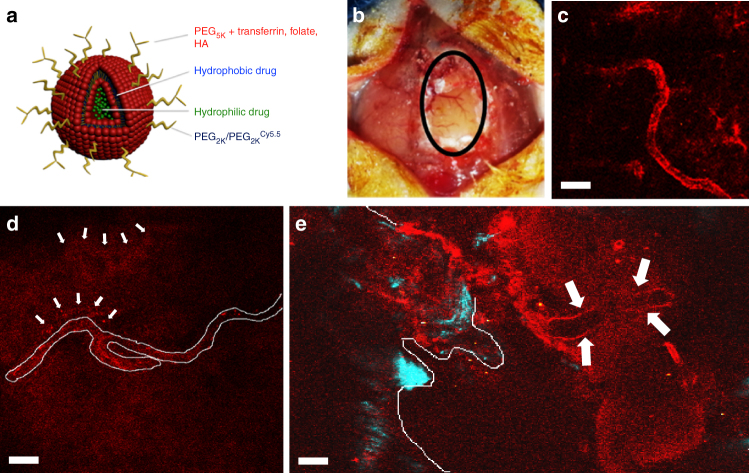
Transferrin-functionalized nanoparticles cross the intact BBB. **a** Schematic of a PEGylated dual drug-loading liposome that can be functionalized to enhance transport across the BBB and targeting to glioma cells. **b** Cranial window (black oval delineating region of craniotomy) exposing the brain for in vivo multiphoton imaging. Multiphoton images of: **c** A brain microvessel showing lack of transport of Hemagglutinin-PEG_2K_-Cy5.5 liposomes across the BBB; **d** Diffusion of transferrin-PEG_2K_-Cy5.5 (Tf-NP) liposomes across the endothelium of a brain microvessel (outlined in white) with nanoparticle aggregates in the subarachnoid space (white arrows); and **e** Composite image showing accumulation of Tf-NP liposomes in the endothelial wall of a brain microvessel (white arrows) with diffusion across the BBB and aggregation of liposomal nanoparticles in the surrounding brain milieu. White outline depicts bony edge of the cranial window with bone second harmonic signal in blue. Images were taken 24 h following a single tail vein injection of nanoparticles. All scale bars = 25 μm

Current Food and Drug Administration (FDA)-approved nano-sized liposomal drug carriers of cancer therapies such as Doxil® largely depend on passive enhanced vascular permeation effects to achieve preferential accumulation in tumor tissues^1^. We recently developed a polyethylene glycol (PEG)ylated liposomal nanoparticle (*Z*_avg_ *d*_h_ ~ 130 nm), which, like Doxil®, achieves stability in the circulation, but in addition, can be functionalized via a PEG 2000 Dalton (Da; PEG_2k_) surface linker with molecular targeting ligands such as folate, and has the ability to partition a hydrophobic drug in the lipid envelope while incorporating a hydrophilic drug in the aqueous interior of the liposome for delivery of dual combination therapies in a single nanoparticle (NP), achieving durable uptake by tumors in vivo when delivered systemically (Fig. 1a)^2^. The simplicity and versatility of this NP led us to hypothesize that it may also have the ability to traverse the blood–brain barrier (BBB), which has much greater vascular restrictions and tight endothelial barriers to NP transport (with upper size limit of blood-tumor barrier  pores ~7–100 nm)^3^, if functionalized with molecular ligands that can facilitate transport across the BBB^4^.

Glioblastoma (GBM; WHO Grade IV glioma) is the deadliest primary brain malignancy with a mere median survival of ~15 months with current standard of care therapies^5^. Improving treatment outcomes for GBM have been limited by the inability to deliver most chemotherapies and novel small molecule inhibitors across the BBB, which effectively excludes most molecules >400 Da^6^. Despite enhanced permeability and leakiness of glioma-associated tumor vessels, the heterogeneous distribution of tumor vessels, the paucity of intervening tumor cells in the vicinity of these vessels, and altered cerebrospinal fluid (CSF) dynamics, prevents effective delivery of chemotherapies to tumor cells^7^. To address this challenge, NPs have recently been employed to enhance the delivery of existing and novel therapies across the BBB^8^.

NPs are stable polymeric encapsulation systems that can be used to deliver multiple cargo to the central nervous system (CNS)^9^. Functionalizing the surface of NPs with ligands to protein receptors that are commonly expressed on the surfaces of brain capillary endothelium and glioma cells can facilitate delivery across the BBB via receptor-mediated transcytosis and subsequent tumor targeting. Such ligands include transferrin, folate, lactoferrin, interleukin peptide, and low-density lipoprotein^4^. Intracellular transport vesicles can readily accommodate NPs between 100 and 200 nm in diameter via receptor-mediated transcytosis allowing for versatility and adaptability of a wide range of NP constructs^10^. Our previous work demonstrating durable uptake of folate-functionalized NPs in flank xenograft mouse models of triple-negative breast and non-small cell lung cancers led us to hypothesize that we could adopt this NP platform for the delivery of novel combination therapies within a single liposomal nanoscale carrier to intracranial mouse models of GBM^2^.

Finally, the therapeutic efficacy of cancer nanomedicines not only hinges on their ability to target tumors efficiently, they must be able to sustain stability in the systemic circulation without premature release of payload, avoiding effects of systemic drug toxicity and adverse off-target tissue effects. Surface modification of liposomes with PEG imparts a steric barrier to the NPs that decreases their recognition and clearance by the reticuloendothelial system, imparting “stealth-like” properties, thereby increasing circulation time, allowing for accumulation at the tumor site, and minimizing adverse drug toxicities. This is seen with the PEGylated liposomal formulation of doxorubicin (Doxil®), which greatly reduces the cardiotoxicity of doxorubicin^11^. Similarly, GBM patients treated with temozolomide (TMZ), the standard of care chemotherapy for GBM, develop significant bone marrow suppression^5^. In this study, we show that transferrin-functionalized PEGylated NPs (Tf-NPs) can be used to deliver novel combination therapies across the BBB in two intracranial orthotopic mouse models of GBM. Tumor-bearing mice treated with Tf-NPs loaded with TMZ and the bromodomain inhibitor JQ1 have decreased tumor burden and prolonged survival compared to mice treated with TMZ and JQ1 packaged in non-functionalized NPs or free drug combinations. Furthermore, we show that mice treated with liposome-encapsulated therapies have relative protection from systemic drug toxicity, demonstrating the potential for translation of this nanoscale platform to improve outcomes for patients with CNS tumors.

## Results

### Functionalized NPs can cross the intact BBB in mice

We first performed biodistribution studies in non-tumor-bearing mice using NPs that were functionalized with a 1,2-distearoyl-*sn*-glycero-3-phosphoethanolamine-*N*-[amino(polyethylene glycol)-2000] (DSPE-PEG_2K_) linker conjugated with Cy5.5-Transferrin (Tf-NP) or DSPE-PEG_2K_-Cy5.5-Folate (Fol-NP) to assess their ability to cross the intact BBB. Non-functionalized PEGylated NPs (PEG-NP) or NPs functionalized with DSPE-PEG_2K_-Cy5.5-Hemagglutinin (Hg-NP) served as negative controls. We chose to use Cy5.5 fluorescence imaging, understanding that it was a semi-quantitative technique for assessing biodistribution of NPs compared to radio-isotope labeling, to simply demonstrate as proof of concept that the conjugated NPs were able to be transported across intact BBB. Mice given I.V. Tf-NPs demonstrated 1.7% total uptake of the injected dose in the brain, compared to 0.9% in Fol-NP-treated mice, respectively, with negligible accumulation in mice injected with PEG-NP or Hg-NPs, 24 h following the injections (Supplementary Fig. 1A). Quantification of confocal microscopy images through fresh frozen coronal brain sections showed highest accumulations of Tf-NPs in the brain compared to Fol-NPs, with no significant increases in Cy5.5 fluorescence signal detected using Hg-NPs compared to unconjugated PEG-NPs (Supplementary Fig. 1B,C). This provided further preliminary evidence that the functionalized NPs can cross the intact BBB in mice.

We then performed multiphoton intravital live imaging through a cranial window (Fig. 1b) to assess the ability of these NPs to cross the BBB in non-tumor bearing mice. To control for vessel leakiness, we injected 70 kDa FITC-Dextran intravenously to ensure vessel integrity following the cranial window procedure (Supplementary Movie 1). Autofluorescence was controlled by increasing signal to noise ratio, thus all Cy5.5 red fluorescence seen in this and subsequent intravital images represents the presence of Cy5.5-labeled NPs only. As Tf-NPs demonstrated the highest percent uptake in the brain and had the smallest average diameter (*Z*_avg_ *d*_h_ ~ 137 nm) (Table 1), we decided to conduct the remainder of our experiments using Tf-NPs. As a negative control, we injected Hg-NPs intravenously and failed to demonstrate uptake by the endothelium of brain microvessels (Fig. 1c). In contrast, Tf-NPs demonstrated transport across the endothelium of microvessels into the surrounding subarachnoid space (Fig. 1d). This was further appreciated upon imaging 500 μm deeper into the cortical mantle, where diffusion of Tf-NPs was observed across an isolated section of a microvessel, forming a diffusion gradient of NPs away from the blood vessel (Fig. 1e).

**Table 1:** Average diameter, polydispersity index, zeta potential, and drug-loading properties of liposomes

Nanoparticle	*Z*_avg_ *d*_h_ (nm)	Polydispersity index	Zeta potential (mV)	JQ1 loading (wt%, EE)	TMZ loading (wt%, EE)	JQ1 + TMZ loading (wt%, EE)
PEG	136	0.15	−6.3	N/A	N/A	N/A
Tf-PEG	137	0.17	−12.1	2.1%, 35.1%	2.9%, 48.3%	JQ1: 2.3%, 38.3% TMZ: 2.7%, 45.1%
Fol-PEG	165	0.11	−13.7	N/A	N/A	N/A
Hg-PEG	160	0.13	+7.6	N/A	N/A	N/A

Average diameters (*Z*_avg_ *d*_h_, nm) and polydispersity indices (PDI) of PEGylated-(PEG) liposomes or liposomes functionalized with transferrin (Tf), folate (Fol), or hemagglutinin (Hg). Drug-loading properites of Tf-NPs containing either JQ1, Temozolomide, or JQ1 + Temozolomide (wt% = % of nanoparticle mass that is drug; *EE* encapsulation efficiency)

### Gliomas take up transferrin-functionalized NPs

**Fig. 2:**
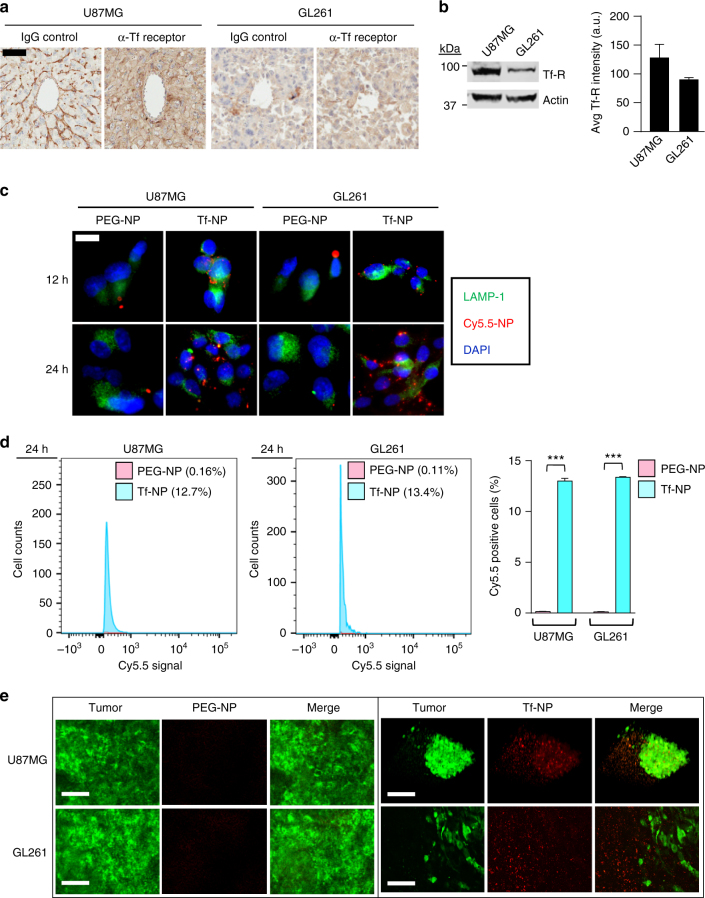
Transferrin-functionalized liposomes achieve receptor-mediated transcytosis and delivery to intracranial models of GBM. **a** Immunohistochemistry demonstrates expression of transferrin receptor (α-Tf receptor) in the endothelium of tumor-associated blood vessels and in tumor tissue of U87MG and GL261 glioma brain tumors. Mouse IgG served as a negative control for non-specific antibody staining (IgG control). Scale bar = 20 μm. **b** Representative western blot and quantification shows ~1.4-fold increased expression of transferrin receptor in U87MG compared to GL261 cells. Data presented as mean ± SEM of three separate experiments. **c** Immunofluorescence staining demonstrates time-dependent intracellular uptake of Tf-NPs but not PEG_2K_-Cy5.5 (PEG-NP) liposomes in U87MG and GL261 cells in vitro. Tf-NPs co-localize to late endosomal/lysosomal compartments (LAMP-1). Nuclei were visualized using DAPI counterstain (DAPI). Scale bar = 10 μm. **d** Flow cytometry plots and quantification of cellular PEG-NP or Tf-NP signal in U87MG and GL261 cells. Data presented as mean ± SEM of three separate experiments. Statistical analysis performed using Student’s *t*-test (***p < 0.001). **e** Multiphoton images of PEG-NP or Tf-NPs (red) at the site of GFP-expressing U87MG and GL261 intracranial gliomas (green). Scale bars = 6.25 μm or 12.5 μm

We next assessed the ability of Tf-NPs to achieve receptor-mediated transcytosis in two intracranial orthotopic mouse models of gliomas: the human U87MG and murine GL261 glioma models. Immunohistochemistry (IHC) staining demonstrated transferrin receptor expression in the endothelium of tumor-associated blood vessels and on tumor cells in both U87MG and GL261 tumor-bearing mice, with relatively higher intensity of staining in U87MG compared to GL261 tumors (Fig. 2a; α-Tf Receptor). This increased uniform staining for the transferrin receptor throughout the tumor was not seen in serial sections stained using control IgG antibody (Fig. 2a; IgG Control). Western blot analysis also further demonstrated ~1.4-fold increased expression of transferrin receptor in U87MG compared to GL261 cells, consistent with the relative differences in staining intensity observed between tumor types on IHC (Fig. 2b). Our findings are consistent with studies demonstrating transferrin receptor expression in U87MG^12^ and GL261^13^ cells, suggesting that transferrin would be suitable as a ligand for our NPs for assessing receptor-mediated transcytosis and tumor targeting as previously reported in the literature^14, 15^. We first assessed the ability of U87MG and GL261 cells to internalize Tf-NPs in vitro. Cells incubated with Cy5.5-Tf-NPs showed increased intracellular Cy5.5 signal which co-localized to late endosomal/lysosomal compartments compared to cells incubated with Cy5.5-PEG-NPs, visualized using immunofluorescence microscopy over the course of 24 h (Fig. 2c). This intracellular uptake was then quantified using flow cytometry, demonstrating an average of 13% Cy5.5-positive cells after 24 h of incubation with Cy5.5-Tf-NPs, compared to <1% Cy5.5-positive cells after incubation with Cy5.5-PEG-NPs (Fig. 2d). These results suggest that functionalization with transferrin is required for cellular uptake of NPs in U87MG and GL261 glioma cells.

We then assessed the ability of Tf-NPs to achieve delivery to brain tumors in vivo. Tumors were allowed to grow for 14 days post implantation. Tumor-bearing mice were treated with an I.V. injection of Tf-NPs on day 14 post-tumor induction and their tumors subjected to multiphoton imaging 7 days following I.V. injection (tumor induction day 21). Multiphoton imaging demonstrated accumulation and uptake of Tf-NPs on the surface of U87MG (Fig. 2e, upper right panels & Supplementary Movie 2) and GL261 tumors (Fig. 2e, lower right panels & Supplementary Movie 3), demonstrating the ability of Tf-NPs to achieve accumulation and retention on the surface of intracranial gliomas. This was in stark contrast to non-functionalized NPs (PEG-NPs) which failed to demonstrate accumulation or uptake by U87MG and GL261 tumors (Fig. 2e, left panels), suggesting that transferrin functionalization is required for transport across the BBB and delivery of NPs to glioma-bearing mice.

### TMZ and bromodomain inhibitor therapy is additive in gliomas

**Fig. 3:**
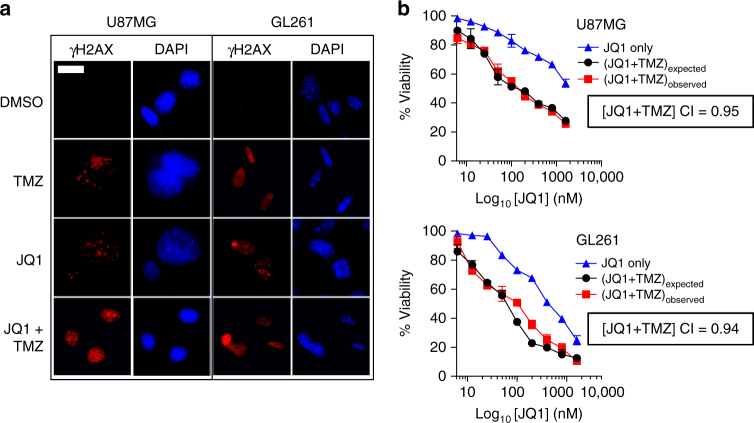
The bromodomain inhibitor JQ1 and temozolomide have additive effects in U87MG and GL261 glioma cells. **a** Representative immunofluorescence images of γH2AX DNA damage foci in U87MG and GL261 cells treated with 500 nM JQ1 and/or 150 μM TMZ for 72 h. Scale bar = 10 μm. **b** Cell viability plots demonstrating combinatorial effects of JQ1 and TMZ. Combinatorial index (C.I.) values determined using the Chou–Talalay method. Data presented as mean ± SEM of three separate experiments

Current gold standard of care treatment for gliomas in humans includes the DNA damage-inducing alkylating agent TMZ^5^. We recently published the novel observation that inhibition of BET bromodomain proteins with the potent small molecule inhibitor JQ1 regulates the DNA damage response in multiple cancer cell lines in vitro, including U87MG cells^16^, and others have also reported antitumor effects following BET bromodomain inhibition in mouse models of glioma^17^. As gliomas show rapid resistance to TMZ^18^, we hypothesized that the addition of a bromodomain inhibitor may further sensitize gliomas to TMZ therapy. To demonstrate sensitivity of the glioma cell lines to bromodomain inhibition, we first treated U87MG and GL261 cells with 500 nM JQ1 for 48 h and observed increased γH2AX DNA damage foci formation in cells (Fig. 3a). DNA damage foci were also seen in cells treated with 150 μM TMZ, with additive effects when cells were treated with both JQ1 and TMZ (Fig. 3a).

To further explore the combinatorial effects of TMZ and JQ1, we performed conventional IC_50_ analyses in U87MG and GL261 cells and calculated combinatorial indexes using these two drugs. Cells treated with incremental log-fold increases in JQ1 in the presence of 150 μM TMZ demonstrated decreased viability quantified using the CellTiter-Glo^®^ viability assay (Fig. 3b, red lines) compared to cells treated with log-fold increases of JQ1 alone (Fig. 3b, blue line). Calculation of JQ1 and TMZ combinatorial index (C.I.) values using the Chou–Talalay method^19^ generated C.I. values of 0.95 and 0.94 for U87MG and GL261 cells, respectively, suggesting that the combination of JQ1 and TMZ achieved additive cytotoxic effects. This data was in line with our observation that single agent JQ1 or TMZ treatment was able to elicit DNA damage in glioma cells, and that the combination of JQ1 and TMZ increased DNA damage over either drug alone (Fig. 3a).

### Tf-NP encapsulated drugs have superior therapeutic efficacy

**Fig. 4:**
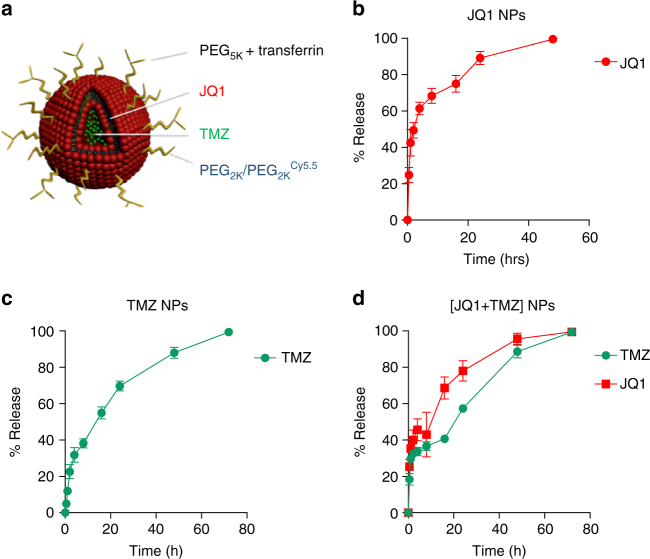
Characterization of drug release from JQ1 and TMZ liposomes. **a** Schematic of liposomes loaded with JQ1 and TMZ. Kinetics of drug release from liposomes loaded with **b** JQ1 alone, **c** TMZ alone, or **d** both JQ1 and TMZ. Data presented as mean ± SEM of three separate experiments

We next interrogated the therapeutic efficacy of drug-loaded NPs compared to free drug dosing in our mouse models of glioma. Despite TMZ and JQ1 both having good penetration across the BBB^20, 21^, only 20% of total serum drug levels of TMZ are achieved in the CSF at best^20^ (a paucity of data regarding the neuropharmacology for JQ1 exists), thus we hypothesized that the ability to package both drugs in NPs for targeted delivery to the site of the tumor would combat CSF washout effects and increase treatment efficacy. To test this, we first packaged TMZ and JQ1 into NPs (Fig. 4a, schematic) and characterized the kinetics of drug release from the NPs. Single or dual drug-loaded NPs were incubated in float-a-lyzer^®^ devices with a 100,000 MW cut-off at 37 °C in normal saline with agitation. Samples were removed from the devices at multiple time points over a course of 72 h and analyzed using high-performance liquid chromatography to quantify drug release from the NPs over time. NPs loaded with JQ1 demonstrated ~90% release of drug by 24 h (Fig. 4b) while NPs loaded with TMZ demonstrated an attenuated release profile with ~90% release by 48 h (Fig. 4c). Dual drug-loaded NPs demonstrated similar release kinetics with relatively more rapid release of JQ1 compared to TMZ and complete release of both drugs by 72 h (Fig. 4d).

**Fig. 5:**
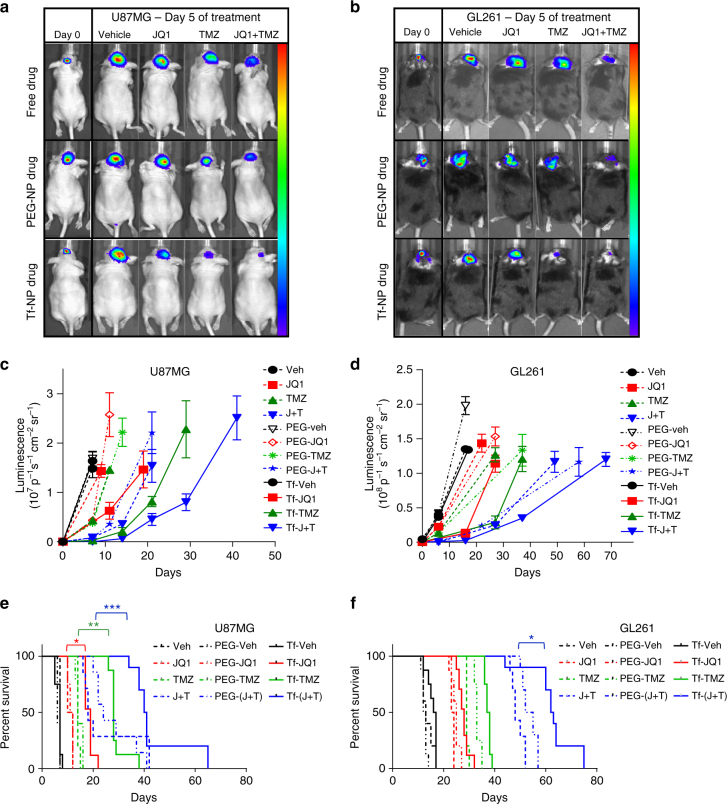
Tf-NPs loaded with JQ1 and TMZ have superior pharmacodynamic effects in intracranial orthotopic models of glioblastoma. Representative bioluminescent images of **a** U87MG and **b** GL261 mice taken on day 0 and day 5 following initiation of treatment with free drug formulations (free drug), drugs loaded in PEG-Cy5.5 liposomes (PEG-NP drug), or transferrin-PEG-Cy5.5 liposomes (Tf-NP drug). Quantification of average tumor bioluminescence values in the different treatment arms throughout the course of treatment for **c** U87MG and **d** GL261 mice. Kaplan–Meier survival plots of **e** U87MG and **f** GL261 glioma mice in the different treatment arms. Study powered with eight mice per treatment arm for statistical significance. Log-rank (Mantel–Cox) test performed on survival plots. Student *t*-test used to quantify differences in treatment arms (*p ≤ 0.05; **p ≤ 0.01; ***p ≤ 0.001)

Tumor-bearing mice were then treated daily with I.V. injections of vehicle, JQ1, TMZ, or JQ1 + TMZ in free drug form, or drugs packaged in either non-functionalized PEG-NPs or Tf-NPs at equivalent doses of 2 mg kg^−1^ per drug for 5 days and tumor signal evaluated using luciferase bioluminescence. Both U87MG (Fig. 5a) and GL261 (Fig. 5b) tumors showed decreases in tumor signal following 5 days of free JQ1 or TMZ compared to vehicle-treated mice, with additive effects when both drugs were combined. Mice treated with drug-loaded PEG-NPs demonstrated similar decreases in tumor signal compared to mice treated with free-drug, however, mice treated with equivalent doses of drugs packaged in Tf-NPs experienced slower longitudinal tumor growth quantified using bioluminescence imaging compared to mice treated with free drug or PEG-NP drug regimens across all treatment arms (Fig. 5c, d). U87MG mice treated with Tf-NP encapsulated JQ1 and TMZ demonstrated a 99.1% decrease in tumor signal compared to an 82% and 79% decrease in signal when treated with free JQ1 and TMZ or dual drug-loaded PEG-NPs, respectively, after 7 days of treatment (Table 2). Similarly, GL261 mice treated with Tf-NP encapsulated combination therapies demonstrated a 99.3% decrease in tumor signal compared to a 97% and 96% decrease in signal in mice treated with either free drug combinations or dual drug-loaded PEG-NPs, respectively (Table 2). These results suggest that functionalization of drug-loaded NPs with transferrin allows for targeted delivery of drugs across the BBB to tumor cells in vivo to achieve significant reductions in tumor burden compared to untargeted drug-loaded NPs, which only achieve reductions in tumor burden similar to free drug regimens.

**Table 2:** Relative fold change in tumor bioluminescence over the course of drug treatment

Cell line	Average fold increase in tumor bioluminescence at day 7 of treatment											
	Free drug				PEG-NP drug				Tf-NP drug			
	Veh	JQ1	TMZ	JQ1 + TMZ	Veh	JQ1	TMZ	JQ1 + TMZ	Veh	JQ1	TMZ	JQ1 + TMZ
U87MG	341 ± 64	280 ± 91	96 ± 27	18 ± 6	430 ± 27	122 ± 7	96 ± 2	21 ± 1	354 ± 26	53 ± 10	7 ± 1	0.9 ± 0.3
GL261	77 ± 16	44 ± 5	25 ± 6	3 ± 2	88 ± 1.8	42 ± 6	25 ± 7	4 ± 0.5	74 ± 5	14 ± 5	18 ± 3	0.7 ± 0.1

Average fold change (±S.D.) in tumor bioluminescence in different treatment arms at day 7 of treatment

Finally, treatment with Tf-NP therapies translated into significantly prolonged survival of U87MG (Fig. 5e; solid lines) and GL261 (Fig. 5f; solid lines) mice compared to mice treated with either free drug (Fig. 5e, f; dashed lines) or drug loaded in PEG-NPs (Fig. 5e, f; dotted lines). These results suggest that the ability of Tf-NPs to achieve receptor-mediated transcytosis across the BBB and accumulate at the site of glioma tumors (Fig. 2e) allows for superior delivery of drugs into tumor cells, compared to free drugs or drugs encapsulated in non-functionalized NPs.

### Tf-NP therapies have increased pharmacodynamic effects in vivo

**Fig. 6:**
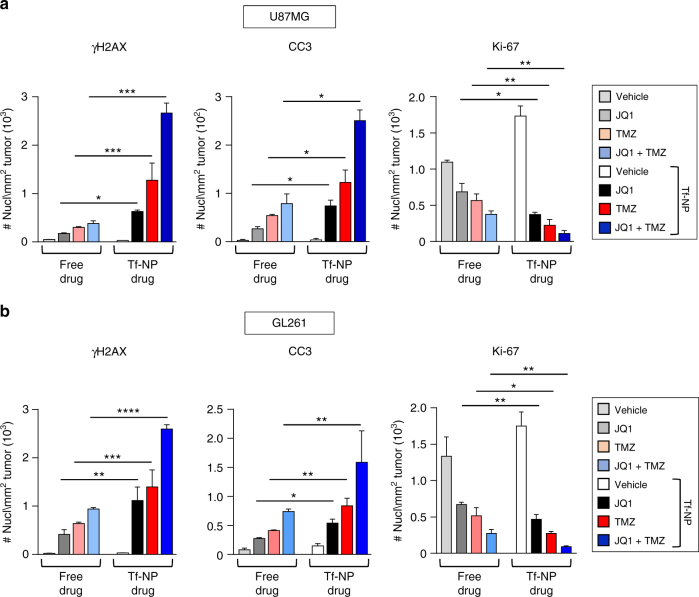
U87MG and GL261 mice treated with dual drug-loaded Tf-NPs demonstrate increased DNA damage and apoptosis in tumors. Quantification of number of tumor cells that stained positive for markers of DNA damage (γH2AX), apoptosis (CC3), and proliferation (Ki67) in **a** U87MG and **b** GL261 mice that received free drug vs liposome-loaded drug (Tf-NP). Signal intensity was quantified from >300 cells in tumors of three mice per treatment condition using ImageJ. Data presented as mean ± SEM (*p < 0.05, **p < 0.01, ***p < 0.001, ****p < 0.0001)

To test the hypothesis that Tf-NPs achieve increased delivery of drugs to tumors compared to free drug regimens, we performed IHC staining of brain sections of U87MG and GL261 mice to quantify the amount of DNA damage and apoptosis in tumors between the different treatment arms. U87MG and GL261 mice were sacrificed 1 week following treatment initiation and brains were sectioned and stained for markers of DNA damage (γH2AX), apoptosis (cleaved caspase 3), and proliferation (Ki-67). Mice treated with drug-loaded Tf-NPs had brain tumors that showed increased staining for γH2AX and CC3 compared to mice that received free drug (Fig. 6 & Supplementary Fig. 2). These increases in markers of DNA damage and apoptosis corresponded with decreased numbers of cells that stained positive for the proliferative marker Ki-67 in both U87MG and GL261 tumors (Fig. 6 & Supplementary Fig. 2), suggesting that our observed binding of Tf-NPs on the surface of tumors led to effective release of drugs into the parenchyma of U87MG and GL261 tumors. Furthermore, tumors from U87MG and GL261 mice treated with dual drug-loaded Tf-NPs showed significantly increased DNA damage and apoptosis with decreased proliferation compared to mice treated with single drug-loaded Tf-NPs (Fig. 6 & Supplementary Fig. 2). These results demonstrate the superior efficacy of combined TMZ and bromodomain inhibitor therapy when packaged in functionalized dual drug-loaded NPs in achieving tumor control and survival in mice with gliomas compared to treatment with equivalent combination free drug dosing.

### NP therapies protect from systemic drug toxicity

**Fig. 7:**
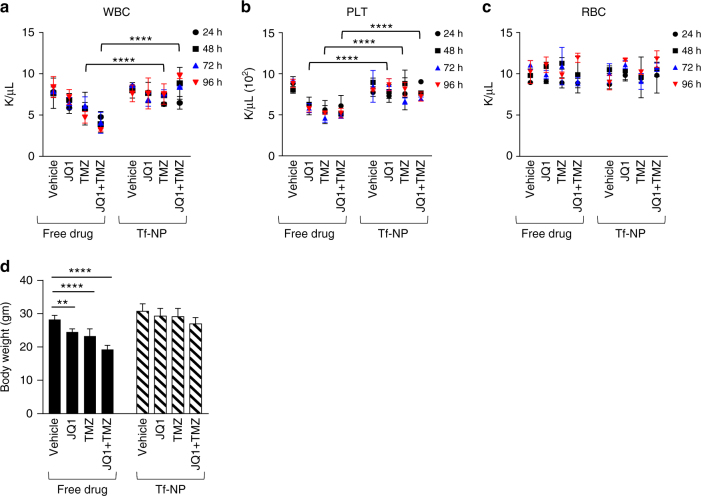
Immunocompetent GL261 mice treated with drug-loaded Tf-NPs are protected from systemic drug toxicity. Mice treated with drug-loaded Tf-NPs demonstrated relative protection from **a** leukopenia and **b** thrombocytopenia caused by JQ1 and TMZ. Mice in either treatment arm did not demonstrate drops in RBC counts (**c**), which is known to be unaffected by JQ1 or TMZ. **d** Quantification of average daily body weights of mice at the end of the 96 h treatment course. Study powered with five mice per treatment arm for statistical significance. Student *t*-test used to quantify changes in hematopoietic profiles between free drug and Tf-NP treatment arms (*p < 0.05; **p ≤ 0.01; ***p ≤ 0.001, ****p ≤ 0.0001)

Finally, we hypothesized that the protective PEGylated packaging and imbued stealth capability of the NPs would offer relative shielding from the known systemic toxicities of JQ1^22^ and TMZ^23^. To test this, we performed serial daily blood monitoring of red blood cell (RBC), white blood cell (WBC), and platelet (PLT) levels in immunocompetent BL6 mice throughout the treatment period comparing values between mice treated with free drug versus Tf-NP-loaded drug. Mice treated with free JQ1, TMZ, or JQ1 + TMZ had progressive leukopenia (Fig. 7a) and thrombocytopenia (Fig. 7b) over the 5-day course of treatment. In contrast, mice treated with drug-loaded Tf-NPs had significantly more stable WBC and PLT levels over the treatment course, suggesting that the PEGylated exterior of the NPs protected the mice from drug toxicity effects. There were no significant changes in the red blood cell counts in either treatment arms (Fig. 7c). Furthermore, mice treated with drug-loaded Tf-NPs maintained their body weight and body conditioning scores compared to mice treated with free drug, which demonstrated significant weight loss and poor body conditioning (Fig. 7d). Although we did not perform liver function tests, we extrapolated that the lack of weight loss and maintained body conditioning in mice treated with drug-loaded NPs was an indication of normal liver function despite the NPs having a high degree of tropism for the liver as shown in our biodistribution studies. This data supports the ability of these PEGylated NPs to compartmentalize drug and retain it in the circulatory system in stealth mode while achieving targeted therapeutic effects in the brain.

## Discussion

To the best of our knowledge, this is the first report comparing the efficacy of combined TMZ and bromodomain inhibitor therapy in multiple models of GBM using a ligand-targeted liposomal nanocarrier. Studies have shown therapeutic efficacy using daunorubicin-loaded liposomes functionalized with transferrin and p-aminophenyl-α-d-manno-pyranoside in C6 rat intracranial orthotopic tumors^24^, or transferrin modified cyclo-[Arg-Gly-Asp-d-Phe-Lys]-paclitaxel conjugate loaded micelles^25^ and doxorubicin-loaded liposomes dual functionalized with a D-peptide ligand of the acetylcholine receptor and cyclic-[Arg-Gly-Asp] peptide^26^ in U87MG intracranial orthotopic tumors, however, neither daunorubicin, paclitaxel, nor doxorubicin are first-line therapies for GBM. Transferrin-functionalized magnetic silica poly(d,l-lactic-co-glycolic acid) NPs have been used recently to deliver doxorubicin and paclitaxel simultaneously to intracranial orthotopic U87MG tumors, however the application of this technology is limited due to FDA restriction on human exposure to magnetic fields^27^. Our transferrin functionalized, dual-drug loading liposome system effectively delivers FDA-approved TMZ and a bromodomain inhibitor (which is currently in clinical trials for several different types of cancers, including glioma^28^) to tumors in the brain with the ability to enhance survival and reduce systemic toxicity, reflecting the translational potential to use this platform to deliver novel combination therapies to improve treatment resistance in patients across multiple CNS tumor types.

Brain capillary endothelial cells express receptors for transferrin, folate, insulin, leptin, low-density lipoprotein, and insulin-like growth factor to allow for transport of essential nutrients including transferrin, glucose, and insulin to maintain brain homeostasis^15^. Malignant tumor cells in the brain also express transferrin and folate receptors, amongst others, on their cell surfaces^4, 29, 30^. For these reasons, these ligands have been widely exploited for receptor-mediated transcytosis of nanotechnology across the BBB for targeted delivery to brain tumors^31^. IHC staining of U87MG and GL261 brain tumors demonstrated transferrin receptor expression in tumor-associated blood vessels and tumor cells (Fig. 2a) and intravital imaging showing uptake of Tf-NPs into the endothelium of brain microvessels (Fig. 1e) and at the tumor site (Fig. 2e), suggests that functionalization of our NPs with transferrin allows for receptor-mediated transcytosis despite having an average diameter of 140 nm. Furthermore, the lack of accumulation of non-functionalized NPs at the tumor site suggests that functionalization with transferrin is required for delivery of the NP across the BBB to the site of the tumor (Fig. 2e). We further postulate that the increased therapeutic response in the U87MG model over the GL261 model is likely due to the approximately 1.4-fold increase in transferrin receptor expression in U87MG cells compared to GL261 cells (Fig. 2b), allowing for enhanced retention of Tf-NPs at the site of U87MG. This also points to the need for careful screening of surface receptor protein levels in patients with gliomas at the time of biopsy or surgical resection in order to achieve a personalized approach to ligand-targeted nanotherapy.

Bromodomain inhibitors are currently in different phases of preclinical and clinical trials for the treatment of multiple tumor types^32^. Two recent studies have demonstrated in vitro and in vivo efficacy of JQ1^17^ and an orally bioavailable bromodomain inhibitor OTX015^33^ in murine models of glioma. Cheng and colleagues reported apoptosis and decreased levels of Myc, Bcl-2, and Bcl-xL following JQ1 treatment in primary patient-derived glioma xenografts in nude mice^17^. Berenguer-Daize et al. reported single agent efficacy of JQ1 or OTX015 alone or in combination with TMZ, everolimus, or SN38 in U87MG, T98G, and U118 GBM cell lines, and in heterotopic and orthotopic U87MG xenografts in nude mice^33^. Interestingly, a common theme that emerged from both studies is that inhibition of Myc following bromodomain inhibition does not appear to factor into the anti-tumor mechanism of drug action in glioma cells. This is further supported by the observation that JQ1 does not reduce BRD4 occupancy at the *MYC* enhancer in U87MG cells^34^. Our observations that treatment of U87MG and GL261 cells with JQ1 leads to increased markers of DNA damage and apoptosis both in vitro (Fig. 3a) and in vivo (Fig. 6 & Supplementary Fig. 2) suggest that this novel mechanism of DNA damage following bromodomain inhibition can be harnessed in combination with other DNA damage repair pathway inhibitors to increase cell death across multiple tumor types. Finally, despite the anti-tumor effects of bromodomain inhibitors, its potent systemic toxicity profile may ultimately limit its use in the clinic. The ability of a targeted nanocarrier to shield against its toxicity may help overcome this hurdle to improve its safety and efficacy.

## Methods

### Chemicals and reagents

(+)-JQ1 was purchased from Cayman Chemicals. TMZ was purchased from Sigma. DOPG and POPG were purchased from Avanti Polar Lipids. DSPE-PEG_2K_/end-functionalized DSPE-PEG_2K_-(NH_2_/maleimide) was purchased from Nanocs, Inc. Cholesterol, sodium, sodium carbonate, and solvents (chloroform, methanol, phosphate buffered saline) were purchased from Sigma.

### Liposome preparation

Liposomes were formulated at a mass ratio of 56:39:5 (DSPC:Cholesterol:POPG). (+)-JQ1 was dissolved along with these three components (weight ratio to total lipid ratio = 3:50) in a 2:1 mixture of chloroform:methanol. A thin film of these materials was generated by rotary evaporation at 40 °C at 150 mbar and dessicated overnight until completely dry. Hydration of the lipid film was conducted at 65 °C under sonication in 300 mM citrate buffer (pH 4) for 1 h. Functionalization of liposomes with DSPE-PEG_2K_-folate (Fol-NP), DSPE-PEG_2K_-Hg_10K_ (Hg-NP), or DSPE-PEG_2K_-transferrin (Tf-NP) and DSPE-PEG_2K_-Cy5.5 was conducted using a post-fabrication, post-insertion technique in which micelles of the components (5 mg functional lipid:50 mg total lipid) were incubated with the drug-loaded liposomes under sonication at 65 °C for 30 min followed by filtration through a 0.2 μm PES (polyethersulfone) syringe filter. TMZ was added in a 0.9% sodium chloride solution to load through a pH gradient method. The dispersed solution was sonicated at 65 °C for 5 min to facilitate solubilization of TMZ. The final drug-loaded system was exchanged into PBS (pH 7.4) following centrifugal filtration (100 K MWCO Millipore) to remove citrate and sodium carbonate and any unloaded drug. Empty (lacking drug) liposomes were formulated in the same fashion. Zeta potential measurements were used to confirm the presence of targeting moieties on the surface of the PEGylated base liposome. No precise quantification of number of targeting groups on the surface of the NP was conducted.

### Liposome characterization and drug release studies

Dynamic light scattering and zeta-potential analysis were conducted in 10 mM sodium chloride at 25 °C using a Malvern ZS90 zeta-sizer. High-performance liquid chromatography (HPLC; Agilent technologies) was used to validate drug loading of (+)-JQ1 (*λ*_abs_ = 260 nm, elution ~ 10 min) and TMZ (*λ*_abs_ = 260 nm, elution ~ 2 min). Drug-loaded NPs were then placed inside float-a-lyser devices with a 100,000 MW cut-off (Spectrum Labs) and immersed into normal saline at 37 °C with agitation. Samples were collected at 0, 0.5, 1, 2, 4, 8, 16, 24, 48, and 72 h and percent drug remaining in the NPs was determined using HPLC as described above. Data are presented as mean ± SEM for three separate experiments.

### Biodistribution studies

NCR nude mice (Taconic) were used for biodistribution studies. To attenuate gut fluorescence, an alfalfa-free special diet (AIN-93M Maintenance Purified Diet from TestDiet) was fed to mice 1 week prior to and during experimentation. Cy5.5-labeled PEG-, Hg-, Fol-, or Tf-conjugated liposomes suspended in PBS were administered via tail vein injection. Recovered Cy5.5 fluorescence in necropsied organs was measured using bioluminescence in vivo imaging (IVIS, Caliper Instruments) with LivingImage™ software (Xenogen), performed at 24 h post injection and reported as % injected dose per gram tissue (%ID).

### JQ1 and TMZ sensitivity assays

For immunofluorescence of γH2AX DNA damage foci, U87MG and GL261 cells were grown on glass coverslips coated with poly-l-lysine (Gibco). Cells were exposed to 500 nM JQ1, 150 μM TMZ, or combination of both JQ1 + TMZ for 48 h, then washed three times with PBS and fixed using 4% paraformaldehyde for 20 min. Cells were blocked with goat serum in Triton X-100 and PBS for 1 h and incubated in γH2AX antibody (EMD Millipore, Catalog No. 05-636) at 1:1000 dilution overnight at 4 °C. Coverslips were washed three times in PBS and incubated in secondary Alexafluor antibody (Invitrogen, Catalog No. A11001) and DAPI counterstain (ThermoFisher, Catalog No. 62248) both at 1:1000 dilution for 1 h at room temperature. Coverslips were mounted onto glass slides using ProLong Gold Antifade mountant (ThermoFisher) and images were taken using a Nikon Eclipse 80i fluorescence microscope.

### JQ1 and TMZ C.I. studies

For JQ1 and TMZ C.I. assays, cells were seeded onto 384-well plates in triplicate repeats and treated with a combination matrix of concentrations of JQ1 ranging from 0 to 1.6 μM and TMZ ranging from 0 to 1.25 mM for 72 h. Cell viability was measured using CellTiter-Glo® assay and results normalized to wells containing DMSO vehicle control. C.I. values were calculated using the Chou–Talalay method with CompuSyn software. Data are presented as mean ± SEM for three separate experiments.

### In vitro NP uptake studies

U87MG and GL261 cells were grown on glass coverslips coated with poly-l-lysine (Gibco). Cells were incubated at 37 °C with PEG-Cy5.5-NP or Tf-Cy5.5-NP for 12 and 24 h. Cells were washed three times with PBS before fixing with paraformaldehyde and processed for immunofluorescence using LAMP1 (Abcam, Catalog No. ab24170) as a lysosomal marker and DAPI (ThermoFisher, Catalog No. 62248) as a DNA stain both at 1:1000 dilutions. For quantification of intracellular uptake by flow cytometry, cells were harvested at 24 h following incubation with PEG-Cy5.5-NP or Tf-Cy5.5-NP, washed once in PBS and fixed in 4% paraformaldehyde and PBS with 1% BSA. Cells were washed three times in PBS with 1% BSA. Ten thousand events were recorded and gated for Cy5.5 signal on a FACS Canto II flow cytometer. Percent Cy5.5 positive cells were quantified using FlowJo® software. Data are presented as mean ± SEM from three biological replicates. Statistical significance determined using Student’s *t* test (*p < 0.05, **p < 0.01, ***p < 0.001).

### Quantification of Cy5.5-labeled liposomes in the brain

NCR nude mice were given 200 μL of Cy5.5-labeled PEG-, Hg-, Fol-, Tf-, or CTX-conjugated liposomes suspended in PBS via tail vein injection and their brains harvested 24 h post injection. Five micron thick fresh frozen coronal sections were prepared and stained with DAPI (ThermoFisher, Catalog No. 62248) prior to fluorescence imaging of Cy5.5-labeled liposomes (Cy5.5 channel excitation, 640 nm; emission, 700 nm) and DAPI-stained nuclei (excitation, 360 nm; emission, 460 nm) using a Nikon A1R Ultra-Fast Spectral Scanning Confocal Microscope (Nikon Instruments Inc.). Average integrated fluorescence intensity of Cy5.5-labeled liposomes in the brain was quantified using ImageJ. Statistical significance determined using Student’s *t* test (*p < 0.05, **p < 0.01, ***p < 0.001).

### Intracranial tumor implantation and monitoring

All animal experimentation was performed in compliance with relevant ethical regulations in adherence with the National Institutes of Health (NIH) Guide for the Care and Use of Laboratory Animals and received institutional approval from the Committee on Animal Care at MIT. U87MG human glioma cells were purchased through ATCC and maintained in DMEM media (Gibco) with 10% FBS (Hyclone). GL261 mouse glioma cells were a gift from Dr. M. Hemann and were maintained in DMEM (Gibco) with 10% FBS (Hyclone), 1× Glutamine (Gibco), and 1× Pen/Strep (Sigma). All cell lines were tested for mycoplasma and subjected to IMPACT testing for pathogens prior to use in experiments. Cell lines were transduced with a lentiviral *pLMP-GFP-Luc* vector to allow for stable expression of eGFP and firefly luciferase prior to implantation. Six-week-old NCR nude (Taconic) or C57/BL6 male mice (Taconic) were used to generate intracranial orthotopic U87MG or GL261 gliomas, respectively. In brief, mice were anesthetized using 2% isoflurane and their heads immobilized in a stereotactic headframe using atraumatic ear bars. A burr hole was made using a steel drill bit (Plastics One, Roanoke, VA, USA) 1.4 mm right of the sagittal and 1 mm anterior to the lambdoid suture. Tumors were allowed to grow for 14 days prior to commencement of treatment. Intracranial tumor growth was monitored in vivo using bioluminescence IVIS^®^ imaging (Xenogen, Almeda, CA) equipped with LivingImage™ software (Xenogen). Mice were randomly separated into each treatment arm: (i) Free drug vehicle (DMSO in 5% β-cyclodextran in 0.9% sodium chloride), (ii) Free (+)-JQ1, (iii) Free TMZ, (iv) Free (+)-JQ1 plus TMZ, (v) Tf-NP loaded with vehicle, (vi) Tf-NP loaded with (+)-JQ1, (vii) Tf-NP loaded with TMZ, (viii) Tf-NP loaded with (+)-JQ1 plus TMZ, (ix) PEG-NP loaded with vehicle, (x) PEG-NP loaded with (+)-JQ1, (xi) PEG-NP loaded with TMZ, or (xii) PEG-NP loaded with (+)-JQ1 plus TMZ. Mice were treated 14 days following tumor implantation daily with 100 μL I.V. of free drug or drug-loaded NPs daily for 5 days and tumor response to treatment was tracked every 3–5 days using IVIS imaging. Mice were given 150 μL I.P. of 30 mg mL^−1^ d-luciferin (PerkinElmer) dissolved in PBS 10 min prior to IVIS imaging. Signal intensity was quantified within a region of interest using LivingImage™ software. Eight mice were used per treatment arm to ascertain statistical significance.

### Multiphoton intravital imaging through a cranial window

To fashion a cranial window, the skull was thinned away using a sterile stainless steel 2 mm diameter cylindrical drill bit attached to a high-speed hand drill until the underlying dura mater was exposed. Multiphoton imaging was performed on an Olympus FV-1000MPE multiphoton microscope (Olympus Americas, Waltham, MA) using a ×25, N.A. 1.05 water objective. Excitation was achieved using a DeepSee Tai-sapphire femtosecond pulse laser (Spectro-Physics, Santa Clara, CA) at 840 nm. The emitted fluorescence was collected by PMTs with emission filters of 425/30 nm for Collagen 1, 525/45 nm for GFP-labeled tumor cells and 668/20 nm for Cy5.5 NPs. Collagen 1 was excited by second harmonic generation and emits as polarized light at half the excitation wavelength. To control for vessel leakiness, we injected 70 kDa FITC-Dextran intravenously to ensure vessel integrity following the cranial window procedure. Autofluorescence was controlled by increasing signal to noise ratio, thus all Cy5.5 red fluorescence seen in this and subsequent intravital images represents the presence of Cy5.5-labeled NPs only. All images were processed using ImageJ.

### IHC of brain sections

Brains were harvested two days post treatment for IHC studies. Mice were perfused with neutral buffered formalin via cardiac puncture and their brains extracted and left overnight submersed in formalin. Brains were processed in 5 μm thick sections following paraffin embedding and IHC performed to detect transferrin receptor (ThermoFisher, Catalog No. 13-6800), γH2AX (Cell Signaling Technology, Catalog No. 9718S), cleaved caspase 3 (Cell Signaling Technology, Catalog No. 9664S), and Ki67 (Abcam, Catalog No. ab16667). Mouse IgG (Santa Cruz, Catalog No. sc-2025) was used as a control to assess specificity of transferrin receptor staining. All antibodies were used at 1:200 dilution. Visualization of stained nuclei was performed using DAB staining (Vector Labs, USA). Slides were digitally scanned at ×20 magnification using an Aperio Digital Slide Scanner (Leica) and viewed using the Aperio digital pathology viewing software (Leica). Positively stained nuclei were quantified using ImageJ. Statistical significance determined using ANOVA followed by Tukey’s test (*p < 0.05, **p < 0.01, ***p < 0.001).

### Western blotting

U87MG and GL261 cells were harvested and lysed in low salt lysis buffer (50 mM Tris-HCl, 150 mM NaCl, 1 mM EDTA, 0.5% NP-40, pH 7.4) supplemented with protease and phosphatase inhibitors (Complete mini EDTA-free and PhosSTOP, Roche Applied Science). Normalized samples were loaded onto 4–12% precast Tris-glycine gradient gels (Life Technologies), subjected to SDS-PAGE, and transferred onto 0.2 μm nitrocellulose membranes (BioRad). Immunoblotting was performed using monoclonal antibodies against transferrin receptor (ThermoFisher, Catalog No. 13-6800) and actin (Sigma, Catalog No. A5441) each at 1:1000 dilution. Protein bands were visualized following incubation with Li-Cor fluorescent secondary antibodies (Li-Cor, Catalog No. P/N 925-32212 and P/N 925-68072) at 1:20,000 dilution using a Li-Cor Odyssey Infrared Imaging System (Li-Cor) and quantified using Odyssey software (Li-Cor). Actin was used as a loading control. Protein band intensity quantified using Li-Cor Odyssey software. Intensity of bands averaged over three separate experiments. Uncropped blots are included in Supplementary Information Section.

### Systemic drug toxicity profiling

Serial daily blood sampling was performed throughout the course of drug treatment and specimens analyzed on the day of sample collection independently by the animal testing laboratories at MIT. Daily body weights were recorded throughout the course of drug treatments. Five mice were used per treatment arm to determine statistical significance.

### Data availability

Data supporting the findings of this study are available within the article and the associated Supplementary Information Section. Any other data are available from the corresponding authors upon reasonable request.

## Electronic supplementary material

Supplementary Information

Description of Additional Supplementary Files

Supplementary Movie 1

Supplementary Movie 2

Supplementary Movie 3
