# Supplementary material for: Enhanced efficacy of combined temozolomide and bromodomain inhibitor therapy for gliomas using targeted nanoparticles
Source: Nat Commun. 2018 May 18;9:1991. doi: 10.1038/s41467-018-04315-4 (PMC5959860; doi:10.1038/s41467-018-04315-4)
Supplement: Supplementary file 1 — Supplementary Information [file 41467_2018_4315_MOESM1_ESM.pdf]

“Enhanced Efficacy of Combined Temozolomide and Bromodomain Inhibitor Therapy for Gliomas Using Targeted Nanoparticles”

Supplementary Information

Lam et al.

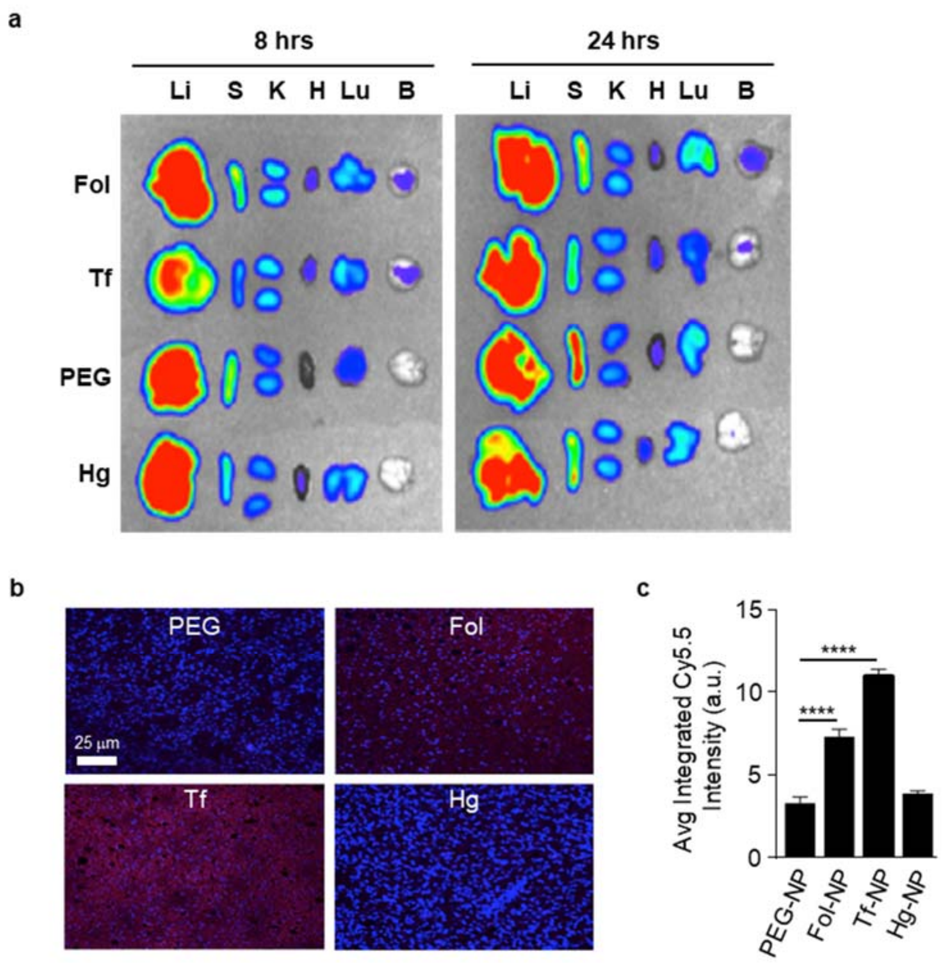

Supplementary Figure 1. Functionalized Liposomes Facilitate Intravascular Transport Across the BBB. a) Representative biodistribution studies demonstrate time-dependent accumulation of folate- (Fol) and transferrin- (Tf), but not hemagglutinin- (Hg) or pegylated- (PEG) liposomes in brains of non-tumor bearing NCR nude mice. **b)** Representative confocal microscopy images demonstrate fluorescence signal in the brains of NCR nude mice 24 hrs following a tail vein injection of Fol- and Tf-functionalized Cy5.5 liposomes but not Hg- or PEG-liposomes. **c)** Quantification of average integrated Cy5.5 liposome fluorescence intensity in the brain slices. Data represents images from 3 mice.

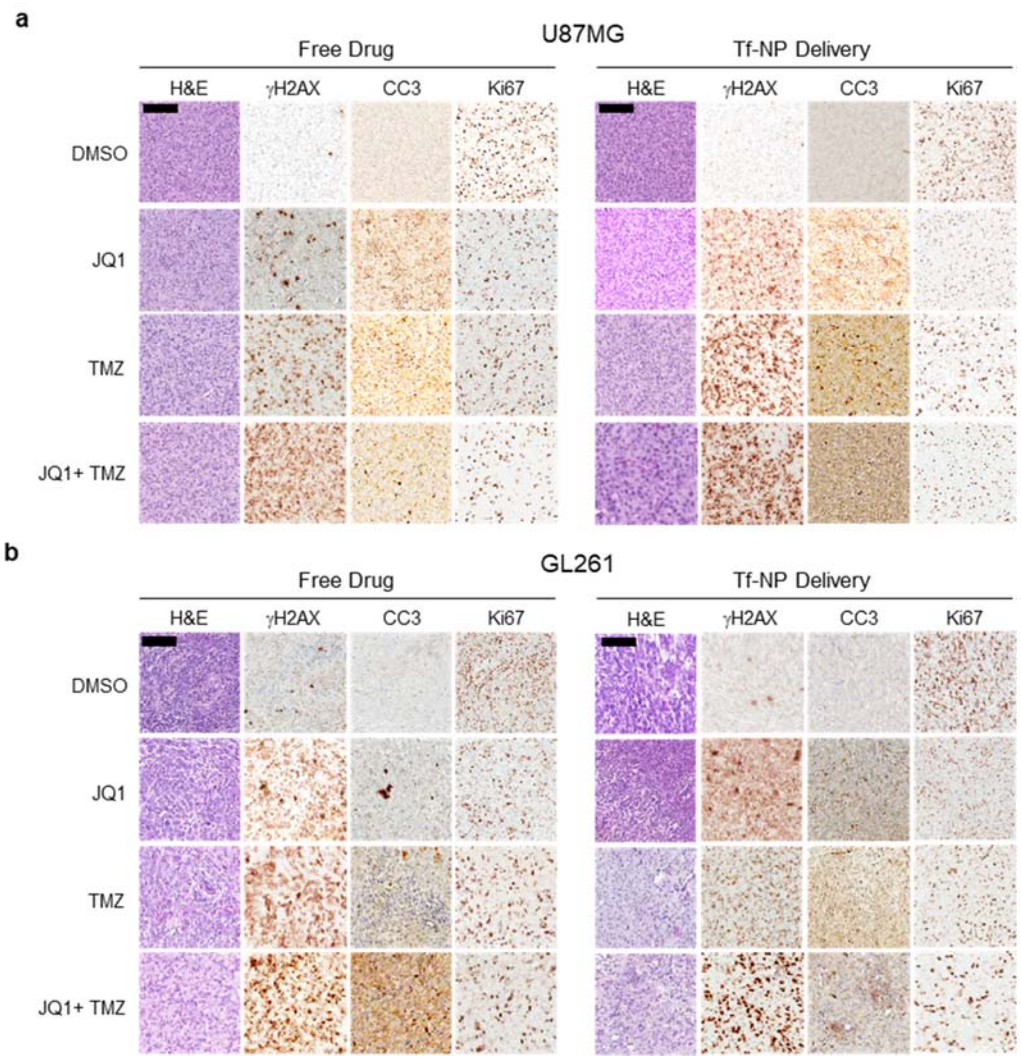

Supplementary Figure 2. Liposome Encapsulated Drug Therapy Achieves Superior *In Vivo* Pharmacodynamic Effects Compared to Equivalent Free Drug Therapy.

Representative immunohistochemistry images of **a)** U87MG and **b)** GL261 intracranial orthotopic gliomas following treatment with either free drug formulations of DMSO, JQ1, TMZ, or JQ1+TMZ; or equivalent doses of liposome encapsulated drug formulations (2 mg kg^{-1}). Slices stained with hematoxylin and eosin (H&E); markers of DNA damage (γH2AX), apoptosis (cleaved caspase 3; CC3), and proliferation (Ki-67). Scale bars = 300 μm.

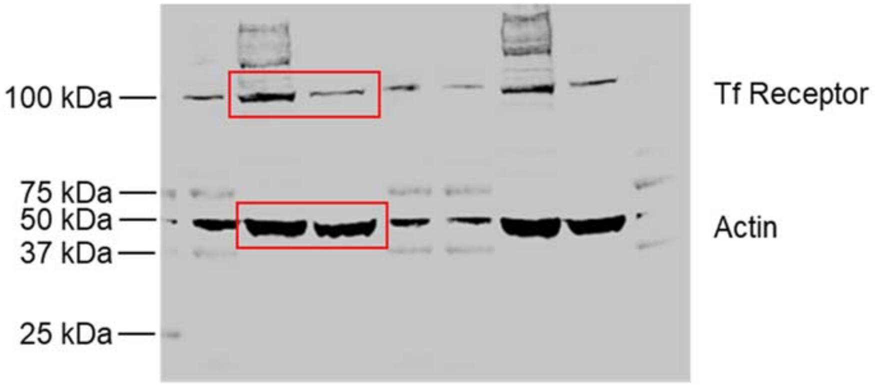

Supplementary Figure 3. Uncropped representative western blot image of transferrin receptor and actin expression from Fig. 2b.

SUPPLEMENTARY MATERIALS AND METHODS

Biodistribution studies

NCR nude mice (Taconic) were used for biodistribution studies. To attenuate gut fluorescence, an alfalfa-free special diet (AIN-93M Maintenance Purified Diet from TestDiet) was fed to mice 1 week prior to and during experimentation. Cy5.5-labelled PEG-, Hg-, Fol-, or Tf-conjugated liposomes suspended in PBS were administered via tail vein injection. Recovered Cy5.5 fluorescence in necropsied organs was measured using Bioluminescence in vivo imaging (IVIS, Caliper Instruments) with LivingImage™ software (Xenogen), performed at 24 hrs post injection and reported as % injected dose per gram tissue (%ID).

Quantification of Cy5.5-labelled liposomes in the brain

NCR nude mice were given 200 μL of Cy5.5-labelled PEG-, Hg-, Fol-, Tf-, or CTX-conjugated liposomes suspended in PBS via tail vein injection and their brains harvested 24 hrs post-injection. Five micron thick fresh frozen coronal sections were prepared and stained with DAPI (ThermoFisher, Catalogue No. 62248) prior to fluorescence imaging of Cy5.5-labelled liposomes (Cy5.5 channel excitation, 640 nm; emission, 700 nm) and DAPI-stained nuclei (excitation, 360 nm; emission, 460 nm) using a Nikon A1R Ultra-Fast Spectral Scanning Confocal Microscope (Nikon Instruments Inc.). Average integrated fluorescence intensity of Cy5.5-labelled liposomes in the brain was quantified using ImageJ. Statistical significance determined using Student's *t* test (*$p<0.05$, **$p<0.01$, ***$p<0.001$).

Immunohistochemistry of brain sections

Brains were harvested 2 days post-treatment for immunohistochemistry studies. Mice were perfused with neutral buffered formalin via cardiac puncture and their brains extracted and

left overnight submersed in formalin. Brains were processed in 5 μm thick sections following paraffin embedding and immunohistochemistry performed to detect transferrin receptor (ThermoFisher, Catalogue No. 13-6800), γH2AX (Cell Signaling Technology, Catalogue No. 9718S), cleaved caspase 3 (Cell Signaling Technology, Catalogue No. 9664S), and Ki67 (Abcam, Catalogue No. ab16667). Mouse IgG (Santa Cruz, Catalogue No. sc-2025) was used as a control to assess specificity of transferrin receptor staining. All antibodies were used at 1:200 dilution. Visualization of stained nuclei was performed using DAB staining (Vector Labs, USA). Slides were digitally scanned at 20X magnification using an Aperio Digital Slide Scanner (Leica) and viewed using the Aperio digital pathology viewing software (Leica). Positively stained nuclei were quantified using ImageJ. Statistical significance determined using ANOVA followed by Tukey's test (* $p<0.05$, ** $p<0.01$, *** $p<0.001$).
