# Supplementary material for: Enhanced efficacy of combined temozolomide and bromodomain inhibitor therapy for gliomas using targeted nanoparticles
Source: Nat Commun. 2018 May 18;9:1991. doi: 10.1038/s41467-018-04315-4 (PMC5959860; doi:10.1038/s41467-018-04315-4)
Supplement: Supplementary file 2 — Description of Additional Supplementary Files [file 41467_2018_4315_MOESM2_ESM.pdf]

Description of Additional Supplementary Files

File Name: Supplementary Movie 1
Description: Assessment of brain vessel leakiness immediately after cranial window surgery. Representative real-time video of brain microvessels taken after cranial window surgery showing flow of intravenous 70 kDa FITC-dextran (green) coursing through microvessels without evidence of leakage.

File Name: Supplementary Movie 2
Description: Transferrin-functionalized Liposomes Accumulate on the Surface of U87MG Tumors. Representative 3D reformatted videos of intravital multiphoton images of U87MG tumors showing accumulation of Tf-NPs (cyan) on the surface of the tumors (green).

File Name: Supplementary Movie 3
Description: Transferrin-functionalized Liposomes Accumulate on the Surface of GL261 Tumors. Representative 3D reformatted videos of intravital multiphoton images of GL261 tumors showing accumulation of Tf-NPs (cyan) on the surface of the tumors (green).
